# Spatial analysis of plague in California: niche modeling predictions of the current distribution and potential response to climate change

**DOI:** 10.1186/1476-072X-8-38

**Published:** 2009-06-28

**Authors:** Ashley C Holt, Daniel J Salkeld, Curtis L Fritz, James R Tucker, Peng Gong

**Affiliations:** 1 Environmental Science, Policy, and Management Department, University of California, Berkeley, CA, USA; 2 California Department of Public Health, Center for Infectious Diseases, Division of Communicable Disease Control, Infectious Diseases Branch, Vector-Borne Disease Section, Sacramento, CA, USA

## Abstract

**Background:**

Plague, caused by the bacterium *Yersinia pestis*, is a public and wildlife health concern in California and the western United States. This study explores the spatial characteristics of positive plague samples in California and tests Maxent, a machine-learning method that can be used to develop niche-based models from presence-only data, for mapping the potential distribution of plague foci. Maxent models were constructed using geocoded seroprevalence data from surveillance of California ground squirrels (*Spermophilus beecheyi*) as case points and Worldclim bioclimatic data as predictor variables, and compared and validated using area under the receiver operating curve (AUC) statistics. Additionally, model results were compared to locations of positive and negative coyote (*Canis latrans*) samples, in order to determine the correlation between Maxent model predictions and areas of plague risk as determined via wild carnivore surveillance.

**Results:**

Models of plague activity in California ground squirrels, based on recent climate conditions, accurately identified case locations (AUC of 0.913 to 0.948) and were significantly correlated with coyote samples. The final models were used to identify potential plague risk areas based on an ensemble of six future climate scenarios. These models suggest that by 2050, climate conditions may reduce plague risk in the southern parts of California and increase risk along the northern coast and Sierras.

**Conclusion:**

Because different modeling approaches can yield substantially different results, care should be taken when interpreting future model predictions. Nonetheless, niche modeling can be a useful tool for exploring and mapping the potential response of plague activity to climate change. The final models in this study were used to identify potential plague risk areas based on an ensemble of six future climate scenarios, which can help public managers decide where to allocate surveillance resources. In addition, Maxent model results were significantly correlated with coyote samples, indicating that carnivore surveillance programs will continue to be important for tracking the response of plague to future climate conditions.

## Background

Plague, caused by the bacterium *Yersinia pestis*, is a disease that has played an important role in human history, most notably through the demographic impacts of three major historical pandemics [1]. Plague was introduced to the United States during the third pandemic (ca. 1900), and spread from the Pacific coast to its current distribution in the western states. Plague is maintained among wild rodents in distinct geographic foci in the western United States [2]. Although the mechanisms by which plague is maintained between epizootic cycles are not well understood, it is generally accepted that the disease cycles between enzootic infections and occasional epizootic outbreaks among susceptible hosts [2]. Humans are presumably at greatest risk of infection during epizootics, when infectious rodent fleas seek a new host. Plague transmission to humans may also occur through contact with infected pets or other animals, through exposure to infected tissue, or via respiratory exposure to infectious air-borne droplets [3,4].

The incidence of human plague cases is relatively low in the United States: for example, a total of 107 cases occurred in the United States from 1990 – 2005 [5], compared to over 240,000 cases of Lyme disease, another vector-borne disease, during roughly the same period (1992 – 2006) [6]. Because of this low incidence, plague surveillance in the western United States is often conducted on a limited budget. However, in contrast to Lyme disease, the case-fatality ratio of plague can be high. If antibiotic treatment is not initiated promptly, plague is fatal in 40–70% of bubonic cases and nearly 100% of pneumonic cases [1]. The combination of low incidence with high mortality presents unique surveillance and public health challenges, because early detection through surveillance may not always be feasible and infrequent clinical cases may be misdiagnosed.

**Figure 1:**
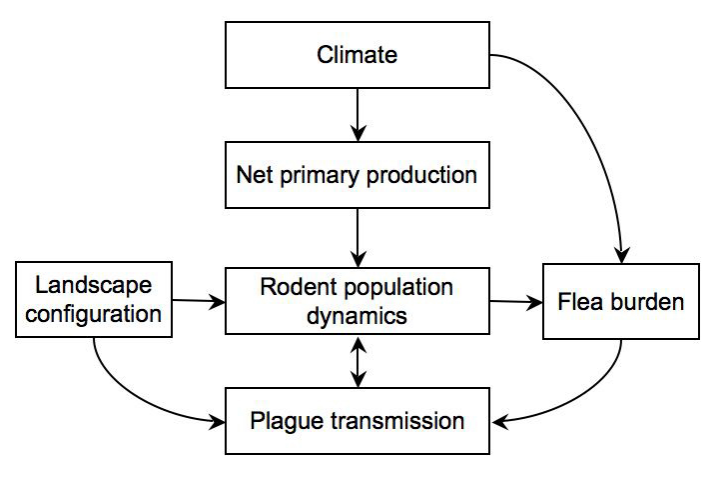
**A conceptual model of the mechanisms by which climate influences plague transmission and maintenance**. Precipitation and temperature have been linked to plague outbreaks in prairie dogs, and to human cases in the United States. A proposed model for this relationship suggests that precipitation and temperature may influence rodent abundance (by influencing rodent survival and food abundance), and that increased rodent populations may affect flea abundance and/or plague transmission rates. In addition to having a positive effect on rodent population dynamics, certain soil moisture, humidity and temperature variables may influence flea ecology and the transmission of the plague pathogen.

In addition, there is concern that certain factors [2,7-10] could increase the occurrence of plague epizootics as well as the risk of exposure and infection to humans. In particular, the direct and indirect effects of climate change on land use, population distribution, and ecologic character are projected to contribute to an increase in the emergence and incidence of infectious diseases [11], including plague. Climate change may drive plague activity through several pathways (Figure 1), including influences on flea burden, rodent population dynamics, and plague transmission [12-19]. A spatially explicit understanding of how plague risk may shift with changing climate patterns can help not only to direct prevention and control efforts, but can also alert health care providers toward quicker recognition of exposure potential and initiation of appropriate treatment of patients [20], which is critical for improving the health outcome of the individual infected as well as reducing secondary transmission to other people.

Recent studies describing the relationships between future climatic and environmental factors and plague activity in the United States have focused on human cases, as well as animal cases in the Southwestern United States and Colorado plateau [12,17,19,21-24]; here, we focus on the potential distribution of plague in California. The point inputs to the models developed in this study were derived from plague serology data collected by the California Department of Public Health (CDPH) and other agencies. Because active surveillance had most often been conducted in areas with a known history of plague-positive rodents or human cases, we used ecological niche modeling (ENM) to identify the potential distribution of plague throughout California (including in previously unsampled areas). Niche modeling has most often been applied to predict the potential for plant and animal species occurrences [for example, [25,26]], and is increasingly being used to identify and map the distribution of diseases, such as Chagas disease [27], filovirus disease [28], Marburg hemorrhagic fever [29], avian influenza [30], and plague [15,31]. In this study we evaluated Maxent, a presence-only niche modeling technique, to describe the potential distribution of plague foci in California under recent and future climatic conditions.

## Methods

### Data

The point inputs to the models developed in this study were derived from plague surveillance data collected by the California Department of Public Health (CDPH) and other agencies [32]. Records of approximately 37,000 animals (33 different genera) collected throughout the state of California during 1984–2004 were entered into an Access database by public health researchers.

#### Rodent point data

Rodent samples were obtained most often by active surveillance, which was conducted in areas with a known history of plague-positive rodents or human cases [32]. Rodent sera were tested by passive hemagglutination to F1 antigen of *Y. pestis*; specimens with antibody titer ≥ 1:32 were considered positive [33-35].

Rodent samples were geocoded based on an address or campsite name, which allowed for location of rodent case point at a <1-km^2 ^spatial resolution. All rodent records were geo-located using National Geographic TOPO software (National Geographic Society 2001). Locations that could not be reliably located to a campground or address were excluded from this analysis. All geocoded points were projected to Teale Albers, NAD 1983 projection. The geocoded locations of the rodent case points are located along the north-south transect of the Sierra Nevada range, and along the southern coast and inland areas of Southern California. No positive rodents were collected in the Modoc plateau, eastern Mojave, or Colorado Desert bioregions during the surveillance period.

**Figure 2:**
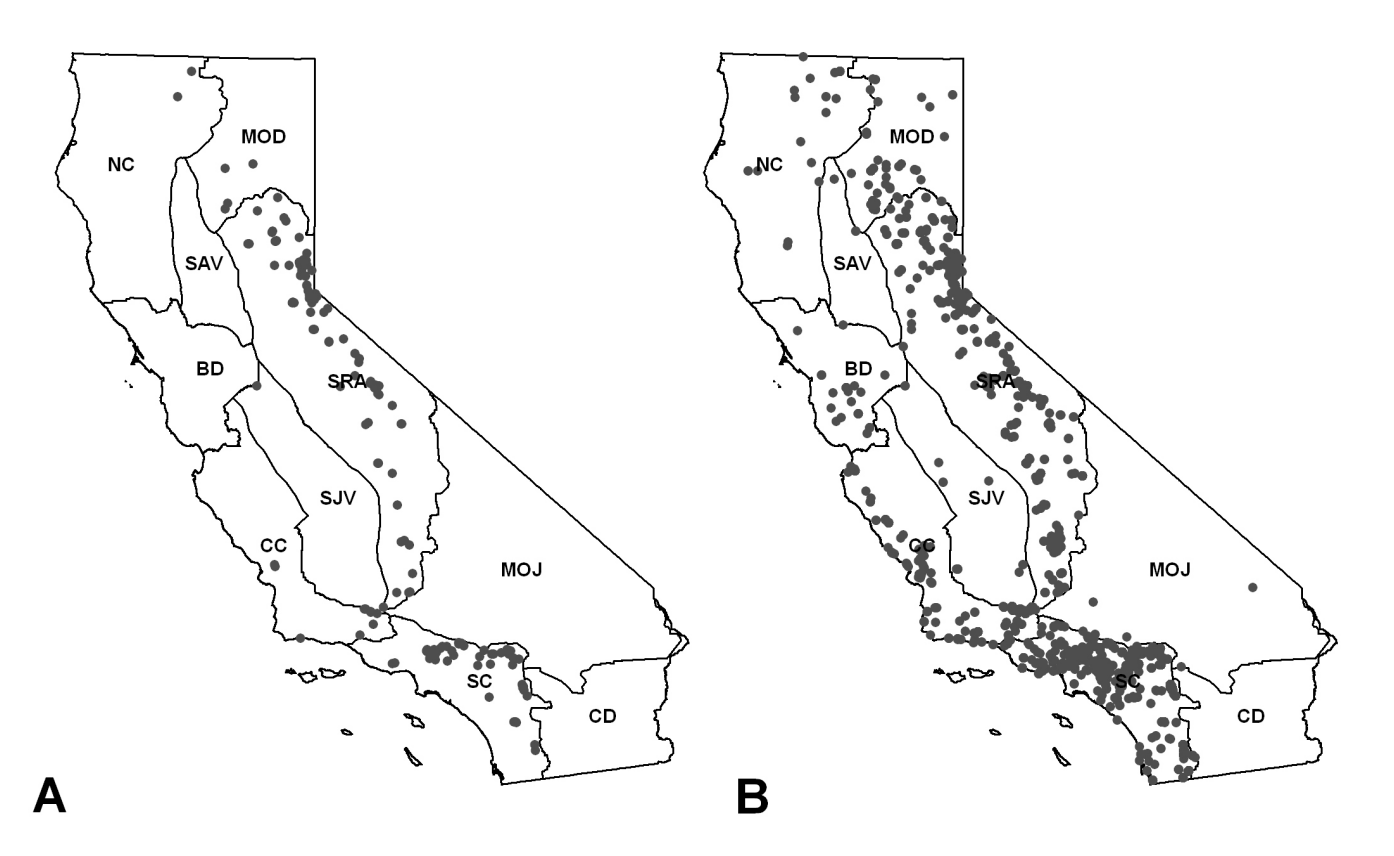
**Rodent samples**. Study area and geocoded data for (a) 995 positive rodent samples (166 unique locations) and (b) 3,788 negative rodent samples (905 unique locations). Lines designate California bioregions (NC = Klamath/North Coast; BD = Bay Area/Delta; CC = Central Coast; SC = South Coast; MOD = Modoc Plateau; SRA = Sierra; SAV = Sacramento Valley; SJV = San Joaquin Valley; MOJ = Mojave Desert; CD = Colorado Desert).

We identified a total of 166 unique locations for positive rodent samples (Figure 2a). The California ground squirrel (*Spermophillus beecheyi*) was the rodent species with the largest total number of specimens (12,546; Table 1) and number of positive specimens (559; Table 1), representing 105 of these unique geocoded locations. Because California ground squirrels are a key indicator species for plague epizootics [36] and human disease risk in California [10], we also ran models for this subset of data only.

**Table 1:** Number of total samples and positive samples for the rodent species most commonly collected during plague surveillance in California

**Species**	**Common Name**	**Total samples**	**Positive samples**	**Prevalence (proportion seropositive)**
*Spermophilus beecheyi*	California ground squirrel	12546	559	0.045
*Tamias senex*	Shadow chipmunk	2701	174	0.064
*Spermophilus lateralis*	Golden-mantled ground squirrel	2685	19	0.007
*Peromyscus maniculatus*	Deer mouse	1776	20	0.011
*Neotoma fuscipes*	Dusky-footed woodrat	1622	10	0.006
*Tamias amoenus*	Yellow-pine chipmunk	1014	58	0.057
*Tamias speciosus*	Lodgepole chipmunk	658	43	0.065
*Tamiasciurus douglasii*	Douglas' squirrel	475	44	0.093
*Spermophilus beldingi*	Belding's ground squirrel	408	21	0.051
*Tamias quadrimaculatus*	Long-eared chipmunk	400	16	0.040
*Neotoma lepida*	Desert woodrat	307	2	0.007
*Tamias merriami*	Merriam's chipmunk	277	18	0.065
*Neotoma cinerea*	Bushy-tailed woodrat	186	6	0.032
*Peromyscus boylii*	Brush mouse	117	2	0.017
*Peromyscus truei*	Piñon Mouse	108	0	0.000
*Tamias minimus*	Least chipmunk	96	2	0.021
*Peromyscus crinitus*	Cañon mouse	82	1	0.012
*Glaucomys sabrinus*	Northern Flying squirrel	71	0	0.000

**Total**		25529	995	0.039

Only records of positive rodents were included for niche modeling, as negative samples (Figure 2b) were frequently obtained from areas that had also yielded positive samples, or from which too few specimens had been collected to be considered representative. 3,788 sampling events had yielded negative samples, but 2,296 of these were at locations where positive samples had also been collected. Of the remaining 1,492 sampling events, only five locations had been sampled more than 20 times, which we estimated as the minimum number of samples that would need to be taken to confirm a location as a true absence.

#### Coyote point data

Sampling for plague in coyotes (*Canis latrans*) was conducted independently from sampling for plague in rodents. Unlike the rodent data, coyote blood specimens were collected opportunistically as part of a depredation control and state-wide plague surveillance partnership between the California Department of Health and the United States Department of Agriculture/Wildlife Services. Because coyotes can occupy a home range of up to 80 km^2 ^[37], the location of capture may not be the location of infection; however, the opportunistic sampling program provides a more complete description of general plague activity throughout the state, albeit at a coarser spatial resolution. Coyote sera were tested by passive hemagglutination to F1 antigen of *Y. pestis*; specimens with antibody titer ≥ 1:32 were considered positive [33-35]. The plague surveillance partnership program and the diagnostic tests that were used are described in detail by [7].

**Figure 3:**
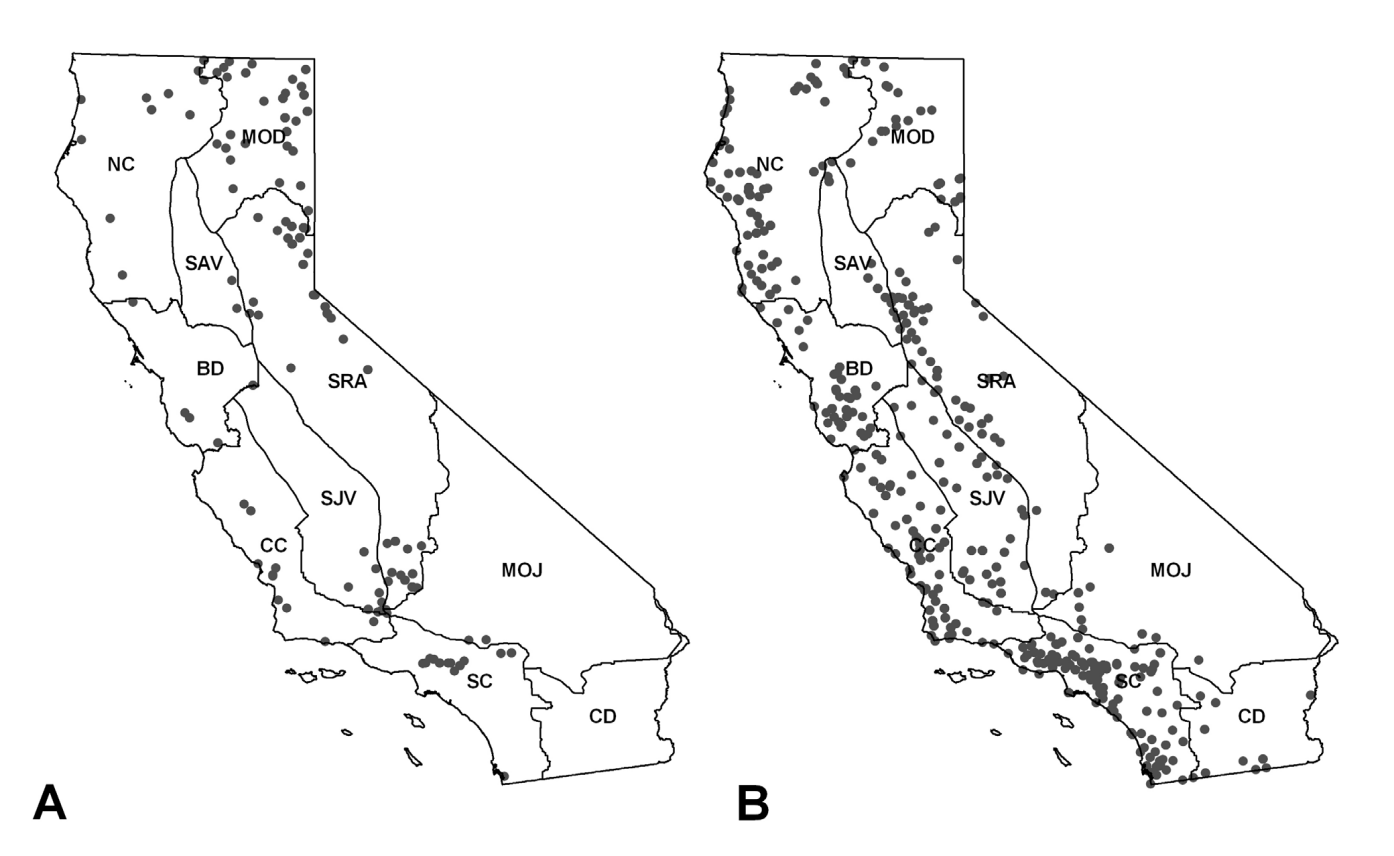
**Coyote samples**. 477 plague-positive coyote samples, and 2,250 negative samples were collected.

In order to compare environmental niche model results based on rodent/ground squirrel data to data on positive and negative samples of California coyotes, records for 477 positive and 2,250 negative coyotes were identified from the database (Figure 3). Collection sites for coyote samples were geocoded using National Geographic TOPO software (National Geographic Society 2001) based on field records that indicated distance and direction from a town [7,33,38]. Collection sites that could not be reliably located were excluded from this analysis. All geocoded points were projected to Teale Albers, NAD 1983 projection. The geocoded locations of the coyote case points were distributed in the northern and southern Sierra Nevada, along the Pacific coast, and across the Modoc plateau.

#### Environmental variables

We downloaded the full set of 19 Worldclim bioclimatic variables  (Table 2). These products are derived from monthly weather station measurements of altitude, temperature, and rainfall. They are biologically meaningful variables that capture annual ranges, seasonality, and limiting factors useful for niche modeling (such as monthly and quarterly temperature and precipitation extremes) [39]. The Worldclim data are at ~1-km^2 ^spatial resolution and have been averaged over a 50-year time period from 1950–2000. Elevation was not explicitly used in model construction because it is already used as a covariate in the Worldclim data production. For modeling purposes, all environmental variable layers were masked to fit the extent of the California state outline. These layers were projected to Teale Albers, NAD 1983 projection.

**Table 2:** Descriptions of BIOCLIM environmental data

**Variable**	**Description**
Bio1	Annual Mean Temperature
Bio2	Mean Diurnal Range (Mean of monthly (max temp – min temp))
Bio3	Isothermality (P2/P7) (* 100)
Bio4	Temperature Seasonality (standard deviation *100)
Bio5	Max Temperature of Warmest Month
Bio6	Min Temperature of Coldest Month
Bio7	Temperature Annual Range (P5–P6)
Bio8	Mean Temperature of Wettest Quarter
Bio9	Mean Temperature of Driest Quarter
Bio10	Mean Temperature of Warmest Quarter
Bio11	Mean Temperature of Coldest Quarter
Bio12	Annual Precipitation
Bio13	Precipitation of Wettest Month
Bio14	Precipitation of Driest Month
Bio15	Precipitation Seasonality (Coefficient of Variation)
Bio16	Precipitation of Wettest Quarter
Bio17	Precipitation of Driest Quarter
Bio18	Precipitation of Warmest Quarter
Bio19	Precipitation of Coldest Quarter

Because the Worldclim variables are derived from a common set of temperature and precipitation data, they can exhibit multicollinearity [39]. A Spearman rank correlation matrix was created in JMP (SAS Institute) to explore the relationships between the Worldclim bioclimatic variables. We removed the four mean temperature variables (Bio8 – Bio11) because they were significantly correlated with minimum and/or maximum temperature variables, and were less likely to be biologically significant in contributing to or limiting plague activity. Of the remaining 15 variables, those that were correlated (Spearman rho > 0.60, p < 0.001) were not used together in the same model. During model runs, a jackknife manipulation was used to assess the relative contribution of each variable, and to remove variables that did not contribute significantly to the model predictions.

### Modeling current and future distribution of plague in California using Maxent

Models of the current potential (i.e. based on climate conditions) distribution of plague in California were run in Maxent (version 3.1.0). Maxent is a machine learning program that uses presence-only data to predict distributions based on the principle of maximum entropy [40]. Maximum entropy [41] is a method to provide the probability distribution which incorporates the minimum amount of information. Given a set of constraints determined by environmental variables or functions thereof, Maxent outputs the maximum entropy distribution that satisfies these constraints. Among species distribution models, Maxent has been shown to provide better identification of suitable versus unsuitable areas when compared to other presence-only modeling methods [40,42]. In place of true absences, Maxent uses background points (pseudo-absences) to evaluate commission.

Maxent does not need multiple model runs to be averaged together [40]; thus, for each set of variables, we ran Maxent once. For each Maxent run, 75% of the points were randomly selected for model training and cross-validation, and 25% of the data were set aside for model testing and independent validation. 10,000 random background points (pseudo-absences) were used to evaluate commission. A regularization setting of 2 was used for data smoothing and to address spatial autocorrelation. Model results were compared and validated using area under the ROC curve (AUC) statistics. The AUC statistic is similar to the Mann-Whitney U test and compares the likelihood that a random presence site will have a higher predicted value in the model than a random absence site [42,43]. One of the appeals of ROC curves is that they do not depend on a user-defined threshold for determining presence versus absence. However, because using a geographical extent that goes beyond the presence environmental domain can lead to inflated AUC scores [44,45], we limited the study area to the rough geographic extent of the sampling distribution (i.e. the California state boundary). The four most predictive models were used as the final models, and mapped as a cumulative probability output.

To explore the spatial relationship between model predictions and serologic samples of carnivores, we compared the final model results to data on positive and negative specimens from California coyotes. We used prediction values extracted for negative and positive coyote specimens using Hawth's point intersect tool [46]. A one-tailed t-test was performed using JMP (SAS Institute) to test the hypothesis that model predictions at positive coyote points would be significantly higher than model predictions at negative coyote points.

In order to simulate the distribution of plague under possible future climate conditions, we ran Maxent using coupled global climate model data from the IPCC 3rd Assessment (available at ). These data were originally produced by three different global climate models: CCCma [47], HadCM3 [48,49], and CSIRO [50], and had been further processed using downscaling procedures in order to match current climate data from Worldclim [39]. We implemented an ArcInfo AML script (freely available at ) to reformat and substantively convert these future temperature and precipitation data into the same bioclimatic variables that had been used as inputs for current-conditions modeling.

For each model we tested for two different time horizons, 2020 and 2050, and two different emissions scenarios (A2 and B2). The A2 scenario assumes that population growth does not slow down and reaches 15 billion by 2100 [51], with an associated increase in emissions and implications for climate change. The B2 scenario assumes a slower population growth (10.4 billion by 2100) and that precautionary environmental practices are implemented [51], yielding more conservative predictions of anthropogenic emissions. To simulate plague response to climate change, we used the final models that had been developed based on the rodent/ground squirrel data, and ran them with the future climate data.

## Results

Four models were selected as the final candidate models predicting plague distribution based on climate variables (Table 3). In all four cases, models based only on California ground squirrel specimens had higher AUC values than their counterpart models that used all rodent samples as case points. Biologically meaningful variables used in these models included two temperature variables (Maximum Temperature of Warmest Month, and Temperature Annual Range) and four precipitation variables (Precipitation Seasonality, Precipitation of Wettest Quarter, Precipitation of Driest Quarter, and Precipitation of Warmest Quarter). The log response charts for the two most important variables used in models of plague in California ground squirrels (Precipitation in the Wet Quarter and Maximum Temperature of the Warmest Month) reflect a quadratic response to increasing temperatures and precipitation (Table 3).

**Table 3:** Maxent final models.

**Model**	**AUC** **(All rodents)**	**AUC** **(*S. beecheyi*)**	**Test omission rate**	**Variables**	**% Contribution**	**Response**	***P***
A	0.876	0.948	0.115	Bio16	47.3	-	< 0.0001
				Bio18	17.6	+	
				Bio15	15.2	+	
				Bio5	13.2	Quadratic	
				Bio7	6.7	+	

B	0.872	0.946	0.115	Bio16	48.7	-	< 0.0001
				Bio5	18	Quadratic	
				Bio17	14.1	Quadratic	
				Bio15	11.9	+	
				Bio7	7.4	+	

C	0.842	.914	0.192	Bio16	56.5	Quadratic	< 0.0001
				Bio5	23.8	Quadratic	
				Bio7	10	+	
				Bio18	9.7	+	

D	0.835	0.926	0.154	Bio16	69.5	Quadratic	< 0.0001
				Bio5	16.5	Quadratic	
				Bio18	13.9	Quadratic	

Test points were used to evaluate omission and 10,000 background points were used to evaluate commission. The reported test omission rate is for equal sensitivity and specificity. The percent contribution of each variable to the models reflects the increase in regularized gain when added to the contribution of the corresponding variable. P-values are for t-test results for the comparison between positive coyote points and negative coyote points (compared by Maxent prediction).

**Figure 4:**
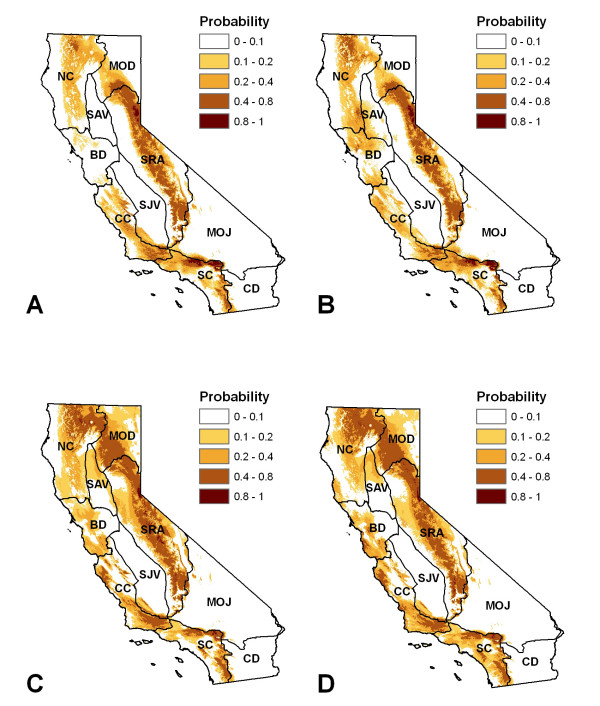
**Maxent model results, using all plague positive rodent samples as case points**. a) Model using Precipitation of Warmest Quarter, Precipitation of Wettest Quarter, Precipitation Seasonality, Temperature Annual Range, and the Maximum Temperature of Warmest Month as predictor variables; b) Model using Precipitation of Driest Quarter, Precipitation of Wettest Quarter, Precipitation Seasonality, Temperature Annual Range, and the Maximum Temperature of Warmest Month as predictor variables: c) Model using Precipitation of Warmest Quarter, Precipitation of Wettest Quarter, Temperature Annual Range, and the Maximum Temperature of Warmest Month as predictor variables; and d) Model using Precipitation of Wettest Quarter, Precipitation of Warmest Quarter and the Maximum Temperature of Warmest Month as predictor variables.

**Figure 5:**
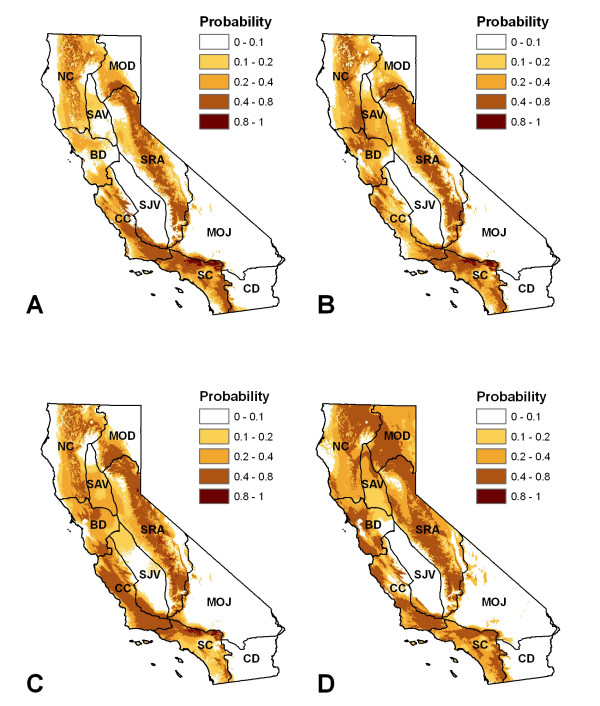
**Maxent model results, using positive California ground squirrel samples as case points**. a) Model using Precipitation of Warmest Quarter, Precipitation of Wettest Quarter, Precipitation Seasonality, Temperature Annual Range, and the Maximum Temperature of Warmest Month as predictor variables; b) Model using Precipitation of Driest Quarter, Precipitation of Wettest Quarter, Precipitation Seasonality, Temperature Annual Range, and the Maximum Temperature of Warmest Month as predictor variables: c) Model using Precipitation of Warmest Quarter, Precipitation of Wettest Quarter, Temperature Annual Range, and the Maximum Temperature of Warmest Month as predictor variables; and d) Model using Precipitation of Wettest Quarter, Precipitation of Warmest Quarter and the Maximum Temperature of Warmest Month as predictor variables.

Models of plague activity in all rodent species (AUC of 0.835 to 0.88) and in California ground squirrels (AUC of 0.913 to 0.948) based on recent climate conditions accurately identified case locations. All models predicted the highest plague activity in the Sierra Nevada and along the southern coast under recent climate conditions (Figure 4 and Figure 5). Models using environmental variables based on squirrel data performed well at predicting plague presence in coyotes. All four Maxent models predicted significantly higher values for pixels that overlapped with positive coyote specimens (Table 3).

**Figure 6:**
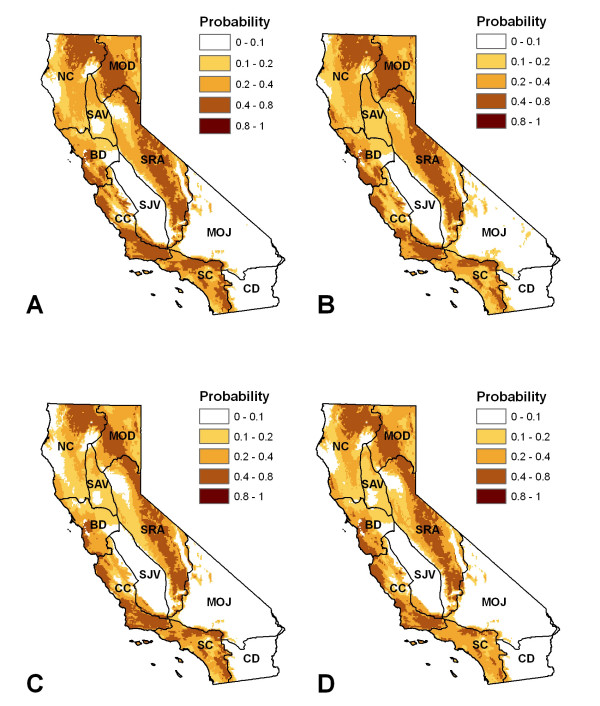
**Predicted future plague distributions**. Models were developed using data derived from three different global climate models (CCCma, HadCM3, and CSIRO), for two time steps and two emissions scenarios. a) 2020, A2 scenario, b) 2020, B2 scenario, c) 2050, A2 scenario, and d) 2050, B2 scenario.

Under future emissions scenarios, our models indicated that climate conditions will drive a) an overall decrease in the probability of plague in the state, b) a subtle shift to higher elevations as well as c) a subtle shift to higher latitudes. Future climate conditions will support increased plague activity in the northern Sierra and central/north coast counties. However, plague risk associated with climate conditions may decrease in the southern Sierras and southern inland counties (Figure 6).

## Discussion

Climate variables, such as temperature, precipitation, and humidity, can play important roles in vector-borne disease transmission by affecting vector and pathogen development, and by influencing the distribution of disease hosts and habitats [11,52]. The biologically meaningful variables that were used in the final models we developed included two temperature variables (Maximum Temperature of Warmest Month, Temperature Annual Range) and four precipitation variables (Precipitation Seasonality, Precipitation of Wettest Quarter, Precipitation of Driest Quarter, and Precipitation of Warmest Quarter). We also found that plague presence exhibits a quadratic response to temperature increases. These results are consistent with other studies [12-14] that have examined the role of temperature and precipitation variables on plague outbreaks in human and animal populations. In addition to having a positive effect on rodent population dynamics, certain soil moisture, humidity and temperature variables may influence flea ecology and the transmission of the plague pathogen [53]. Specifically, while warmer temperatures may in general stimulate plague activity, temperatures above 35 degrees Celsius are associated with a negative effect on flea fecundity, survival, and behavior [13,18,54].

Under future emissions scenarios, our models indicate that climate conditions will drive a) an overall decrease in the probability of plague the state, b) a subtle shift to higher elevations as well as c) a subtle shift to higher latitudes. These results are generally consistent with other climate modeling studies that show species movement to higher latitudes and elevations in response to warming [55], and with studies that have examined the historical record of plague response to climate and show a shift to higher latitudes [16,22]. Several other recent studies have also projected a potential decrease in plague activity in certain areas of the United States in response to more frequent hot days [19,23,24].

In addition, these results provide insight into the relationship between plague maintenance in carnivore and rodent populations. Carnivores, and particularly coyotes, have been implicated in plague transmission and serve as sentinel species for the disease [7,56]. Recent studies [57,58] conducted on the Central Plains Experimental Range and Pawnee National Grasslands (which collectively cover a ~80,000 ha area) link the prevalence of carnivores and rodent hosts in a spatially explicit manner. Our results expand these analyses to a larger scale, by exploring the overlap in predicted plague-positive rodent distributions with positive and negative coyote samples derived through an independent sampling program. Model results demonstrate a link between positive coyote samples and areas of predicted rodent infection, providing additional support for rodent surveillance and follow-up in areas where the carnivore surveillance program identifies plague-positive animals.

California ground squirrels are the rodents that have been the most frequently sampled for plague in California. However, six other species (Douglas' squirrel, Lodgepole chipmunk, Merriam's chipmunk, Shadow chipmunk, Yellow-pine chipmunk, and Belding's ground squirrel) often had higher serum titers than California ground squirrels. This suggests these species may be of interest for further sampling and surveillance, and that additional modeling of these species' distributions could be conducted to explore the spatial heterogeneity of plague foci in California [59]. Maxent models of California ground squirrels fit better than models that used all rodent specimens as training points. Because California ground squirrels occupy a narrower ecologic zone than all rodents collectively, with less variable climatic conditions, these models described a more precise climatic niche for plague.

Current model results matched areas with historical and recent plague activity, including the San Francisco peninsula and San Bruno Mountain, the San Jacinto mountains, and the Los Padres National Forest area [32]. Models did not yield high prediction values for the Modoc plateau region, which has historically been a focus of plague [10]. Because the low population density and rural nature of this area does not readily lend itself to observation of epizootic events, it is not surprising that no positive rodents were collected in the Modoc plateau during the study period. Thus, no model input points were used from this area, which can present a challenge to niche modeling techniques in terms of extrapolating results to new conditions in geographic and ecologic space [60]. In addition, the low prediction values for the Modoc plateau may be related to the extreme climate profile and characteristics of the plague system in this area, where plague maintenance and transmission is driven by a climate regime and rodent-host complex that differ from the rest of California. Many areas of the Modoc plateau experience plague, but in wood rats (*Neotoma *spp.), as well as in yellow pine chipmunks (*Tamias amoenus*) and their associated fleas.

It is important to keep in mind that by modeling the climatic niche for plague in California, we have modeled a potential distribution for plague that is not the actual or realized distribution. Other important factors, including landscape configuration, biotic variables, and barriers to dispersal likely limit the actual distribution of plague to smaller areas than those predicted using a climatic niche modeling approach [40,59,61]. Secondly, a number of studies have demonstrated that different modeling approaches can yield substantially different predictions [42,62]. Thus, future work could include modeling plague potential distributions under a suite of different modeling approaches. Additionally, using niche models to predict distributions into expanded temporal and/or spatial domains can result in significant variance inflation [62]. We have attempted to dampen this variability by averaging future model outputs based on three different global climate models. However, the use of global climate models (as opposed to local or regional climate models) may itself be another source of error in niche modeling studies, and thus a potential area of research could explore the effects of different modeling datasets on disease distributions (for example, see [63]). Finally, averaged climate variables dampen seasonal effects and do not capture climatic anomalies, which may be important drivers of plague epizootics [11-13]. Thus multi-temporal modeling is required to elucidate the effects that increased climatic variability will have on vector-borne disease dynamics.

## Conclusion

Because different modeling approaches can yield substantially different results, care should be taken when interpreting future model predictions. Nonetheless, niche modeling can provide general trends in response to climate conditions. Models of plague activity in California ground squirrels, based on recent climate data, accurately identified plague-positive rodent locations, as well as areas of historical and recent plague activity. Maxent model results were significantly correlated with coyote samples, and suggest that carnivore and rodent plague surveillance programs should be more tightly coupled in California. The final models were used to identify potential plague risk areas based on an ensemble of six future climate scenarios, which can help public managers decide where to allocate scarce surveillance resources.

## Competing interests

The authors declare that they have no competing interests.

## Authors' contributions

ACH, CLF, JRT, and PG designed the study. ACH conducted spatial analysis and Maxent modeling, and contributed to the writing of the manuscript. DJS contributed to the analysis of carnivore data and to the interpretation of model results. CLF and JRT conducted field sampling and collated the data used in this study, and were instrumental in analyzing model results in the context of recent trends in plague activity in California. All authors read and approved the final manuscript.
